# Strengthening Academic Foundations: PINS’ Journey Toward Research Excellence

**DOI:** 10.12669/pjms.41.13(PINS-NNOS).14639

**Published:** 2025-12

**Authors:** Shaukat Ali Jawaid

**Affiliations:** 1 Shaukat Ali Jawaid Chief Editor Pakistan Journal of Medical Sciences Karachi – Pakistan. pjms@pjms.org.pk

Research and publications have historically not been a priority in Pakistan’s medical institutions, nor have they received the attention they deserve until recently. However, when the head of an institution is genuinely committed to academics and eager to promote a research culture, the situation can change significantly. This is exactly what has happened at the Punjab Institute of Neurosciences (PINS). Since assuming responsibility as Executive Director, Prof. Asif Bashir has taken numerous initiatives to strengthen academic activity and research output at the institute.

One of his major contributions has been the establishment of a dedicated Department of Research and Editorial Office, formally inaugurated by his mother, Dr. Almas Bashir, on May 24th, 2025. Under his leadership, several workshops on scientific writing and training sessions were organized for faculty and postgraduate trainees, encouraging them to engage meaningfully in research. As a result, the first supplement of the *Pakistan Journal of Medical Sciences* (PJMS), published last year, successfully showcased the growing body of research emerging from PINS.

This year, PINS advanced its academic efforts even further. A special supplement on Neuro-Oncology was planned in collaboration with the Pakistan Association of Neuro-Oncology (PASNO). In addition to contributions from PINS faculty and postgraduates, numerous researchers from other national and international institutions were invited to submit their work. This strategy proved highly productive, yielding a supplement that covers a wide array of neuro-oncology topics.

Planning for the supplement began in February 2025. As agreed, Prof. Asif Bashir served as the Guest Editor to supervise the 2025 supplement. Dr. Haseeb Mehmood Qadri was again entrusted with the responsibilities of receiving submissions, Letters of Undertaking signed by all listed authors, and ensuring Ethics Committee/IRB approvals. The Secretary to Chief Editor PJMS, Mr. Nouman facilitated plagiarism screening after the initial review and supervised the peer review along with the Guest Editor and Chief Editor PJMS. Original articles and case reports were reviewed by at least three subject experts, while systematic reviews were scrutinized by at least four national and international peer reviewers. Authors were also instructed to fully comply with the International Committee of Medical Journal Editors (ICMJE) authorship guidelines to discourage gift authorship.

Of the 86 submissions received, 40 manuscripts were shortlisted for further processing and subsequently reviewed by the PJMS Editorial Team. Fourteen manuscripts with authorship issues were returned for revision, requiring reduction in the number of authors; five manuscripts needed reference corrections; and four were rejected for not meeting PJMS standards, as they cited journals without any peer-review mechanism. Ultimately, this supplement includes 20 original articles, one review, five systematic reviews, seven case reports, three letters, and three editorials.

In his editorial, Prof. Athar Enam, President of PASNO, highlights several important manuscripts included in this issue, while Prof. Asif Bashir provides valuable background on the academic initiative. At *Pakistan Journal of Medical Sciences*, we are pleased to have partnered with PINS in projecting the academic achievements of this major public-sector institution.

A review of 25 years of neuro-oncology research in Pakistan by Haseeb Mehmood Qadri et al. has revealed that Aga Khan University alone contributed 132 publications—a testament to its strong research heritage.¹

Writing on the clinical landscape, perceptions, and training in surgical neuro-oncology, Syed Haider Hassan and colleagues provide critical insights into clinical exposure and training quality among neurosurgical residents. They highlight gaps in hands-on experience, formal training, mentorship, and access to specialized resources, particularly in spinal neuro-oncology. Limited interdisciplinary collaboration and insufficient institutional support further hinder comprehensive skill development.²

Ahtesham Khizar and colleagues emphasize the importance of collaborative, shared-care models in pediatric surgical neuro-oncology, especially in countries with limited resources and few specialized pediatric neuro-oncologists.³ Momin Bashir and colleagues discuss the cognitive and emotional strain experienced by brain tumor patients, noting that such diagnoses pose profound physical, mental, and financial challenges. Addressing both physical and mental health needs through targeted support can significantly improve patient coping and outcomes.^4^

Rahat Ul Ain and colleagues highlight that primary CNS lymphoma (PCNSL) in children is extremely rare and associated with poor survival rates. However, timely surgical intervention and improved supportive care may help enhance prognosis.^5^

In their study on determinants of poor prognosis after glioblastoma surgery, Sundas Irshad and colleagues conclude that although most cases undergo gross total resection, median survival remains one year. They identify extent of resection as a significant independent predictor of survival, with gross total resection associated with lower mortality.^6^ Overall, this supplement constitutes a valuable addition to the growing body of neuro-oncology literature from Pakistan.

The dedication and commitment of Dr. Haseeb Mehmood Qadri throughout this project has been commendable. We are pleased that both Prof. Athar Enam and Prof. Asif Bashir have recognized and appreciated his efforts. Dr. Qadri, a talented young neurosurgery resident, also received the prestigious Dr. Maqbool H. Jafary Scholarship for his outstanding performance in the Certificate in Medical Editing course at the University of Health Sciences, Lahore. I found him to be an exceptionally capable individual. With his training in the science and art of medical journalism, he can be a tremendous asset to any editorial endeavor. Any professional society or institution could greatly benefit from his expertise, should they choose to engage him. Continued guidance and support from Prof. Asif Bashir will undoubtedly contribute to his professional growth, making him an invaluable resource for managing academic activities at PINS.
